# Dynamic Transcription Machineries in Protocells

**DOI:** 10.1021/jacs.5c03622

**Published:** 2025-05-23

**Authors:** Jiantong Dong, Fan Xia, Fujian Huang, Itamar Willner

**Affiliations:** † The Institute of Chemistry, Center for Nanoscience and Nanotechnology, 26742 The Hebrew University of Jerusalem, Jerusalem 91904, Israel; ‡ State Key Laboratory of Geomicrobiology and Environmental Changes, Faculty of Materials Science and Chemistry, 12564 China University of Geosciences, Wuhan 430074, China

## Abstract

Transcription machineries play key roles in nature by regulating diverse cellular processes, including cell cycle progression, the control of intracellular metabolic balance, and cell differentiation and growth. These processes are regulated by the programmed transcription factor-mediated operation of transcription machineries and cellular environmental cues dictating spatiotemporal gene expression, demonstrating amplification and bistable, switchable, and transient dynamic features. Emulating these native pathways through artificial means not only advances the area of Systems Chemistry by providing principles for the evolution of life but also introduces novel catalytic and theranostic applications of the system. The perspective addresses recent advances in developing transcription-machinery-loaded protocell assemblies, consisting of liposomes, microdroplets, proteinsomes, and microcapsules. Stimuli-responsive transcription machineries integrated into liposomes, Fe^3+^-cross-linked tannic acid membranes, and nucleic acid-functionalized hydrogel microcapsules acting as protocells are triggered by light, redox agents, and switchable refiguration of transcription templates. Moreover, temporally modulated oscillatory transcription circuitries integrated in microemulsion droplets acting as protocells were demonstrated, and the transcription-guided transient assembly and disassembly of DNA nanotubes mimicking formation and dissociation of motor filaments in native cells was accomplished. In addition, the dynamic transcription-mediated diffusive signaling and communication of microdroplets and proteinosome-based protocell assemblies are presented. Future challenges of the topic and potential practical applications of these systems are addressed in the conclusion section.

## Graphical abstract

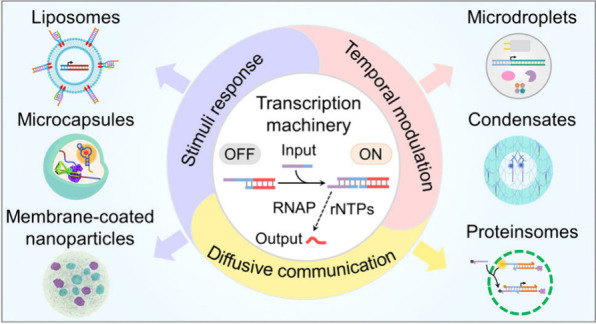

## 1 Introduction

Transcription machineries play key roles in regulating diverse biological transformations, such as cell cycle progression,^1^ control of intracellular metabolic balance,^2^ and cell differentiation, growth, and development.^3,4^ The native transcription apparatus involves an adaptive dynamic machinery modulated by transcription factors programming diverse upstream or downstream gene expression pathways and spatiotemporal biological circuits.^5^ The dynamic regulation of transcription machineries is guided by proximal or remote regulators, such as silencer,^6^ insulators,^7^ or switching elements,^8^ and is eventually dictated by cellular cues, including pH,^9^ nutrient supply,^10^ or stress.^11^ Moreover, the coupling of biological networks and feedback circuits,^12^ or the conjugation of repressing transcription circuits,^13^ yields complex frameworks revealing amplification,^14^ bistable,^15^ oscillatory,^16^ and transient reaction pathways.^17^ In addition, in living systems, transcription and translation occur within spatially confined, membrane-bound cellular microenvironments, where localization and compartmentalization are critical for functionality.^18^ These confined microenvironments support hierarchical organization into cellular and subcell circuits,^19^ enabling tightly controlled biochemical signaling and intercellular communication.^20,21^ As a result, transcription activity contributes to not only intracellular regulation but also adaptive responses to metabolic challenges,^22^ and coordinated behavior across multicellular systems.^23^

Inspired by nature, substantial research efforts are directed to the development of cell-mimicking compartments (protocells) emulating native cellular reservoirs.^24^ While earlier cell-free systems primarily focused on executing basic transcription/translation processes in static environments,^25^ they lacked regulatory mechanisms and responsiveness characteristic of living cells. Bridging this gap requires the development of protocells capable of sensing and responding to environmental cues through temporally controlled gene expression. Incorporating such dynamic regulatory features is essential not only for emulating the dynamic behaviors of natural cells but also for endowing synthetic cell constructs with programmability and adaptivity. Particularly, the integration of synthetic networks and dynamic circuits in protocells attracts growing interest, focusing on the assembly of functional synthetic cells.^26^ Diverse protocell assemblies were introduced, including liposomes,^27^ polymersomes,^28,29^ dendrosomes,^30^ colloidosomes,^31^ proteinsomes,^32,33^ microcapsules,^34^ microdroplets,^35^ and coacervates.^25,36,37^ Different biocatalytic cascades and genetic circuits were integrated into the protocell assemblies, emulating elements of functional living cells.^38^ These advances significantly contributed to the rapidly developing topic of Systems Chemistry.^39–41^ Beyond bridging the gap between synthetic cells and native cells and providing models for the evolution of life, the practical implications of protocells are a continuous scientific holy grail of the field. Indeed, recent efforts highlighted the potential applications of protocells in the areas of catalysis^42^ and theranostics.^43^ The present perspective addresses recent advances in developing dynamic transcription machineries in protocell assemblies, Figure 1. Different protocell compartments, such as liposomes, polymersomes, dendrosomes, membrane-coated porous nanoparticles, proteinsomes, microcapsules, water-in-oil microdroplets, and phase-separated coacervates/condensates (Figure 1A), will be introduced as carriers for the transcription machineries. While all protocell carriers provide compartmentalized environments for transcription reactions, distinguishing them from homogeneous systems^44^ and protecting RNA against enzymatic or chemical degradation, the physical boundaries of these reservoirs play important roles in controlling stability, transport properties, and potential functional applications. For example, membranous protocells, such as liposomes or proteinsomes, often require surface charge modulation or chemical modification to maintain the colloidal stability. Moreover, the incorporation of synthetic transmembrane channels or other interboundary modification is often required to facilitate the transport of agent and interprotocell communication. A range of dynamic transcription machineries integrated into protocell systems, including stimuli-responsive, temporally modulated, and transient (dissipative) systems, will be introduced (Figure 1B). Particularly, stimuli-responsive dynamic transcription processes occurring within these protocell assemblies, emulating native biological transformations, will be presented. This includes the programmable, input-driven operation of transcription machineries triggered by auxiliary stimuli, such as light, pH, redox, ions/ligands, promoters, and transcription factor or inducer analogs, Figure 1C. Transcription circuits exhibiting switchable, gated, cascaded, bistable, or oscillatory (accompanied by feedback loops) behaviors, and transient, dissipative dynamics in the protocells, will be introduced. Specifically, output signals generated by transcription machineries, manifested by changes in optical properties, structural self-assembly, and catalytic activity, will be discussed. Finally, emphasis will be placed on dynamic transcription-mediated communication between protocells.

**Figure 1:**
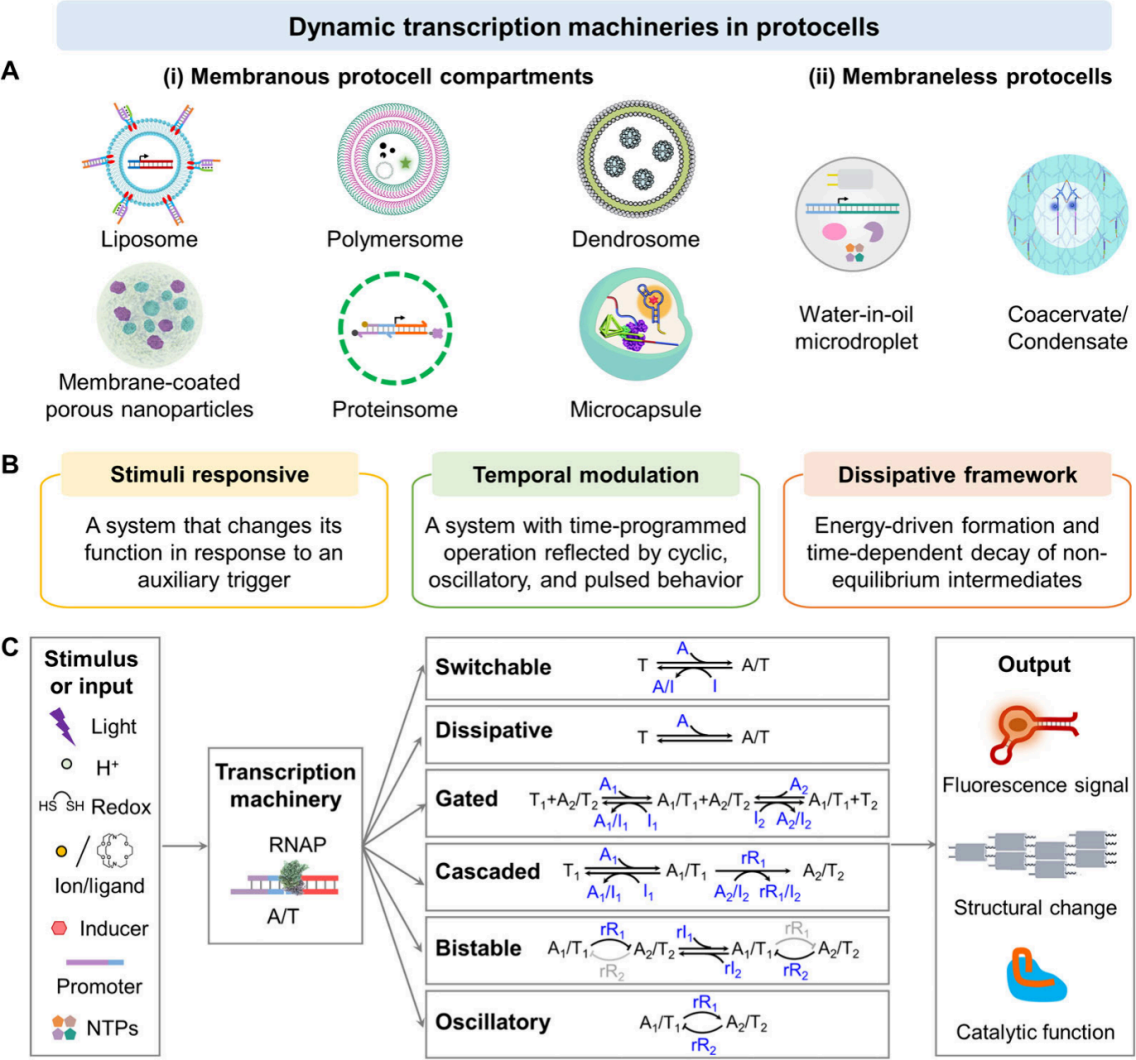
Overview of diverse dynamic transcription machineries in protocells. (A) Schematic display of various protocell architectures, categorized into: (i) membranous protocell compartments and (ii) membraneless protocells. (B) Definition of stimuli responsive, temporal modulation, and dissipative framework assemblies. (C) Schematic depiction illustrating dynamic transcription machineries responding to diverse stimuli or inputs, resulting in signaling, structural, or catalytic outputs.

## 2 Stimuli-Responsive Activation of Transcription Machineries in Protocells

Major challenges related to the stimuli-responsive activation of the transcription machineries in protocell assemblies include the integration of the transcription modules into the protocells, the triggered spatiotemporal activation of the reaction module by auxiliary signals, and the engineering of transducing readout signals to probe the operation of the transcription machinery in the protocell compartments. Different synthetic cell-like protocell compartments, including liposomes, vesicles, and coating membranes, were employed to host the transcription modules.^45^ Incorporation of the machineries into the protocell assembles was aided by membrane transfection means or stepwise fusion of small-sized, partially loaded liposomes with giant liposomes, yielding fused integrated protocells.^46,47^ Activation of the transcription machineries in the protocells was achieved by diverse external triggers, such as light,^48,49^ pH,^50^ or chemical agents.^34,51^ Different physical and spectroscopic tools, such as light scattering, fluorescence, absorbance, and chemical transformations, were employed to characterize the protocell systems and the signal transduction processes proceeding in the protocell compartments.

In a primary report,^48^ liposomes loaded with T7 RNA polymerase (T7 RNAP), a DNA template, the ribonucleotide triphosphates (rNTPs) mixture consisting of GTP, CTP, and UTP, and a photoresponsive caged ATP were used as a protocell model. While the photocaged ATP retained the transcription machinery inactive, the light-induced uncaging of ATP activated the transcription machinery, which then synthesized an RNA product quantified by qPCR. While this study demonstrated the assembly of a synthetic cell-like compartment with light-triggered transcription machinery, the continuous temporal intraliposomal imaging of the transcription process was missing. In a further study,^49^ the stepwise fusion of small liposomes (220 nm in diameter) loaded with subconstituents of the transcription machinery, with a giant liposome (10 μm in diameter) loaded with RNAP and rNTPs, was employed to yield an integrated light- and pH-triggered protocell assembly driving the transcription of the Malachite Green (MG) RNA aptamer, Figure 2A, panel I. The giant liposome N_1_, functionalized at its boundary with the cholesterol-modified photoresponsive *o*-nitrobenzylphosphate ester-caged hairpin (1), was loaded with the RNAP and rNTPs, while the small-sized liposomes N_2_ and N_3_, modified at their boundaries with nucleic acids (2) and (3), were loaded with the transcription template AA’ and MG, respectively. Photochemical uncaging of the hairpin (1) yielded duplex (1)/(1’) that, in the presence of N_2_, led to the displacement of (1’) by (2), resulting in the fusion of N_2_ with N_1_. The subsequent interaction of (3) with the duplex (1)/(2) at pH = 5 yielded the triplex (1)/(2)/(3) interbridged fused liposomes N_1_/N_2_/N_3_. The fusion of the three liposomes led to an integrated cell-like compartment that included all constituents for the operation of the transcription machinery, Figure 2A, panel II. As the A’ component in the transcription template was complementary to the MG aptamer, the operation of the transcription machinery yielded the MG RNA aptamer, forming the fluorescent MG-aptamer complex as the protocell transduction product, Figure 2B. Control experiment revealed that the transcription process was initiated only upon the light (λ = 365 nm) and pH = 5 triggering fusion of the three liposomes N_1_/N_2_/N_3_. Fluorescence confocal microscopy imaging in Figure 2C supported the intraprotocell evolution of the red fluorescence of the MG-aptamer product.

**Figure 2:**
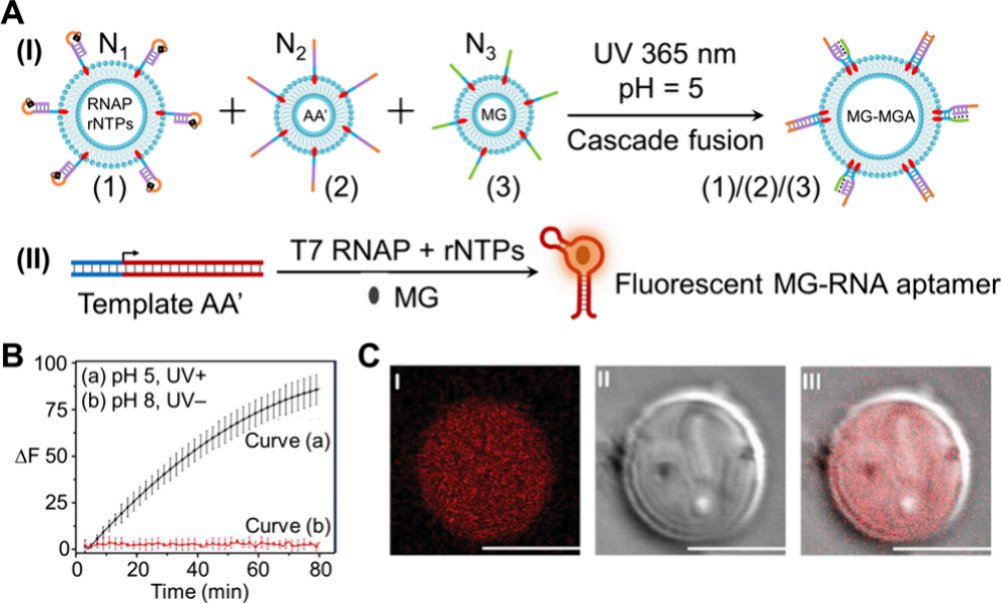
(A) Panel I: Schematic representation of light/pH-induced fusion of three liposomes, N_1_ (loaded with RNAP/NTPs), N_2_ (loaded with the transcription template AA’), and N_3_ (loaded with MG), leading to an integrated protocell liposome containing the intact transcription machinery. Panel II: Transcription machinery-guided synthesis of the fluorescent MG-RNA aptamer in the fused liposome. (B) Temporal fluorescence changes of (a) the transcription machinery-guided synthesis of the MG-aptamer complex in the fused liposome assembly and (b) control system composed of nonfused liposomes N_1_, N_2_, N_3_ in the absence of light/pH activation. (C) Confocal microscopy images of the fused liposomes after 3 h of transcribing the MG-aptamer: panel I – fluorescence image, panel II – bright-field image, panel III – overlaid image of panels I and II. Scale bars, 10 μm. Adapted from ref 49. Available under a CC-BY license. Copyright 2023 The Authors.

In a further example,^50^ pH-sensitive zeolitic imidazolate framework-8 (ZIF-8) metal–organic framework nanoparticles (NMOFs) were employed as carriers to construct protocells loaded with constituents to guide the transcription/translation machineries for synthesizing green fluorescent protein (GFP). Two separate ZIF-8 NMOF carriers were synthesized, where one type of ZIF-8 NMOF (a) included the GFP plasmid, and the second type of ZIF-8 NMOF (b) was loaded with the in vitro transcription and translation (IVTT) constituents, Figure 3A, panel I. The resulting NMOFs were imaged by SEM (panel II). The mixture of the two NMOFs (a) and (b) were integrated in an Fe^3+^-cross-linked tannic acid coating membrane, synthesized according to Figure 3B. To assemble the Fe^3+^-cross-linked tannic acid membrane loaded with the mixture of the two NMOFs, a biphasic system consisting of a water phase (loaded with the NMOFs and tannic acid) was interacted with a hexadecane solution containing iron acetylacetonate (stabilized by a surfactant). Phase transfer of the Fe^3+^ ions to the aqueous phase yielded the cross-linked Fe^3+^-tannic acid polymer loaded with the two NMOFs. The fluorescence images of the Fe^3+^-tannic acid membrane with fluorescent propidium iodide (PI)-labeled ZIF-8 NMOF (a) and Alexa Fluor 350 NHS ester-labeled ZIF-8 NMOF (b) are displayed in Figure 3C. The pH-triggered activation of the transcription/translation machineries in the Fe^3+^-tannic acid cell-like membrane is displaced in Figure 3D. At pH 6.0, the two ZIF-8 NMOFs were degraded, leading to the release of the GFP plasmid from ZIF-8 NMOF (a) and the transcription/translation constituents from ZIF-8 NMOF (b), resulting in the sequestered transcription and translation of the GFP. Figure 3E, panel II, shows the fluorescence images of the GFP generated in the protocell at pH 6.0. As a control system (panel IV), the subjection of the protocell to pH 7.0 did not lead to the transcription/translation of GFP, consistent with the structural stability of the ZIF-8 NMOFs (a) and (b) at this pH value, prohibiting the operation of the transcription/translation process in the protocell.

**Figure 3:**
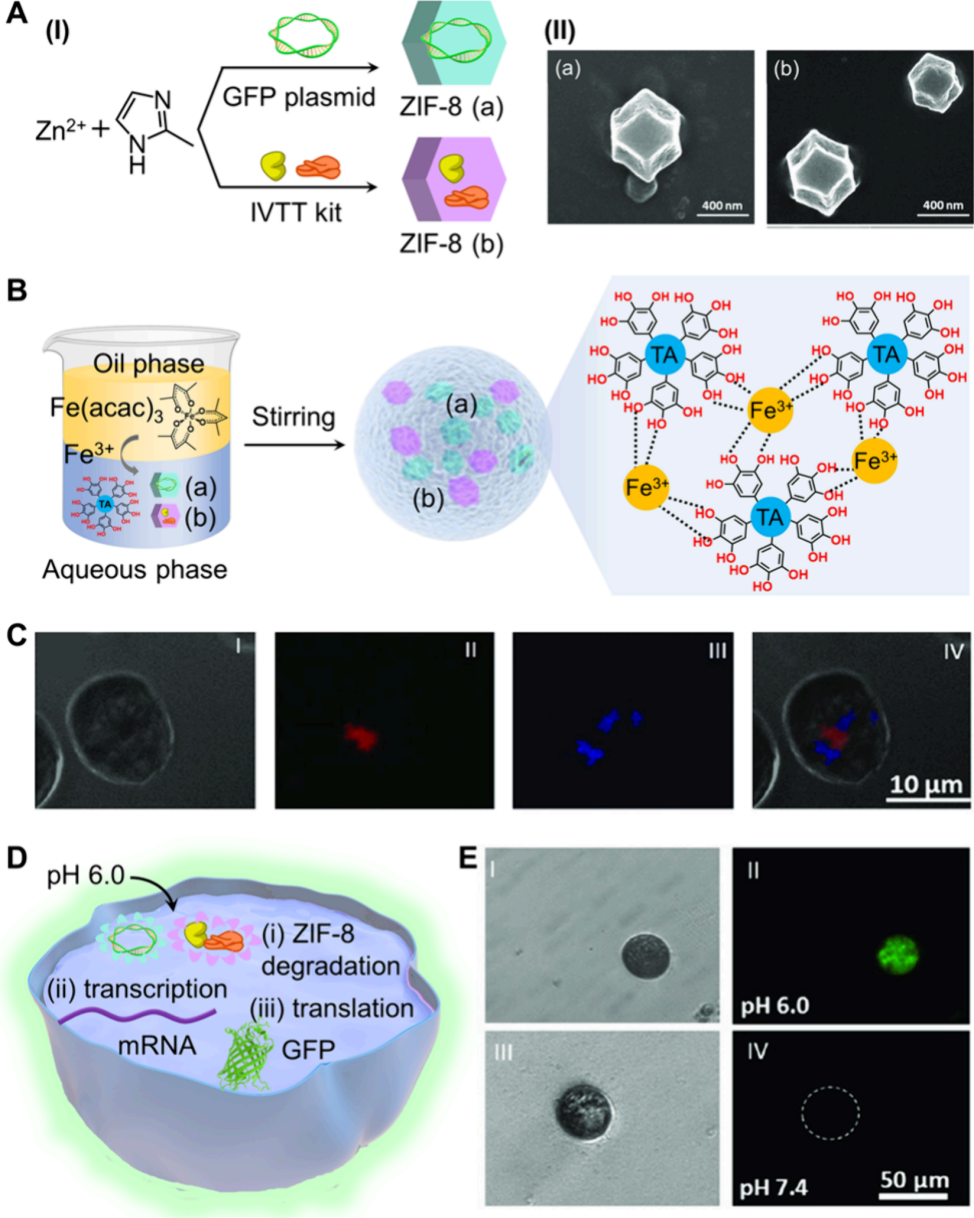
(A) Panel I: Synthesis of ZIF-8 NMOFs encapsulating (a) the GFP plasmid and (b) the in vitro transcription/translation (IVTT) kit. Panel II: SEM images of the ZIF-8 NMOFs loaded with (a) the GFP plasmid and (b) the IVTT kit. (B) Schematic assembly of the Fe^3+^-tannic acid cell-like membrane loaded with a mixture of the two NMOFs. (C) Confocal microscopy images of (I) the artificial cell (bright-field image), (II) the PI-labeled plasmid@ZIF-8, (III) the Alexa Fluor 350 NHS-labeled IVTT@ZIF-8, and (IV) the merged image of I–III. (D) Schematic of pH-stimulated dissolution of the ZIF-8 NMOFs, releasing the transcription/translation machinery in the Fe^3+^-tannic acid cell-like membrane compartments. (E) Confocal microscopy images corresponding to panels I and II: the bright-field image/fluorescent image of the Fe^3+^-tannic acid cell-like compartments after 2 h upon pH 6.0-triggered release of the GFP plasmid/IVTT transcription/translation machinery into the membrane compartments. Panels III and IV: control images for the nonreleased GFP plasmid/IVTT-loaded NMOFs present in the cell-like membrane at pH 7.4. Adapted from ref 50. Available under a CC-BY license. Copyright 2020 The Authors.

Moreover, the redox-triggered thiol-activated deprotection of disulfide-bridged inactive enzyme aggregates (zymogens) into thiol-functionalized active enzymes^52^ has been applied to activate transcription machineries in giant unilamellar vesicles (GUVs) as protocells.^51^ A biphasic system was employed to generate the inactive zymogen transcription machinery in the protocell. This system consisted of an aqueous phase separated from a water-in-oil microemulsion phase containing aqueous microdroplets loaded with the inactive T7 RNAP zymogen (T7-Z_PEG_), the DNA template, and the rNTPs mixture. The phases were separated by an interface that included vesicle-forming constituents (l-α-phosphatidylcholine, cholesterol, poly­(ethylene glycol)-1,2-distearoyl-*sn*-glycero-3-phosphoethanolamine-N, PEG-DSPE) and Rhodamine PE as a dye tracer visualization probe. Stirring the biphasic system resulted in the phase transfer transition of the GUV interphase constituents into the aqueous phase, which incorporated the T7-Z_PEG_, DNA template, and rNTPs from the respective phases into the resulting self-assembled GUVs, Figure 4A. Treatment of the purified protocells with dithiothreitol (DTT) deprotected the T7-Z_PEG_ to yield active T7 RNAP that triggered the operation of the transcription machinery in the protocell, Figure 4B. The resulting GUVs were imaged by the fluorescent tracer (panel I), and the transcribed RNA generated by the transcription machinery was visualized by staining with SYBR Green II (panel II). In a further experiment, a fully blocked transcription machinery consisting of the T7-Z_PEG_, an inactive rNTPs mixture (composed of CTP, GTP, UTP, and adenosine diphosphate, ADP), the transcription template, and the caged creatine kinase zymogen (CK-Z_0_) was loaded in the GUVs, Figure 4C. In the presence of the thiogen and alkaline phosphatase (ALP), thiophenol was formed at the exterior phase of the GUVs and its permeation into the GUVs uncaged the CK-Z_0_ and T7-Z_PEG_, resulting in the CK-catalyzed transformation of ADP to ATP, and activation of the transcription machinery. The operation of the transcription machinery by cooperative deprotection of the two zymogens was then followed by visualization of the transcribed RNA stained with SYBR Green II.

**Figure 4:**
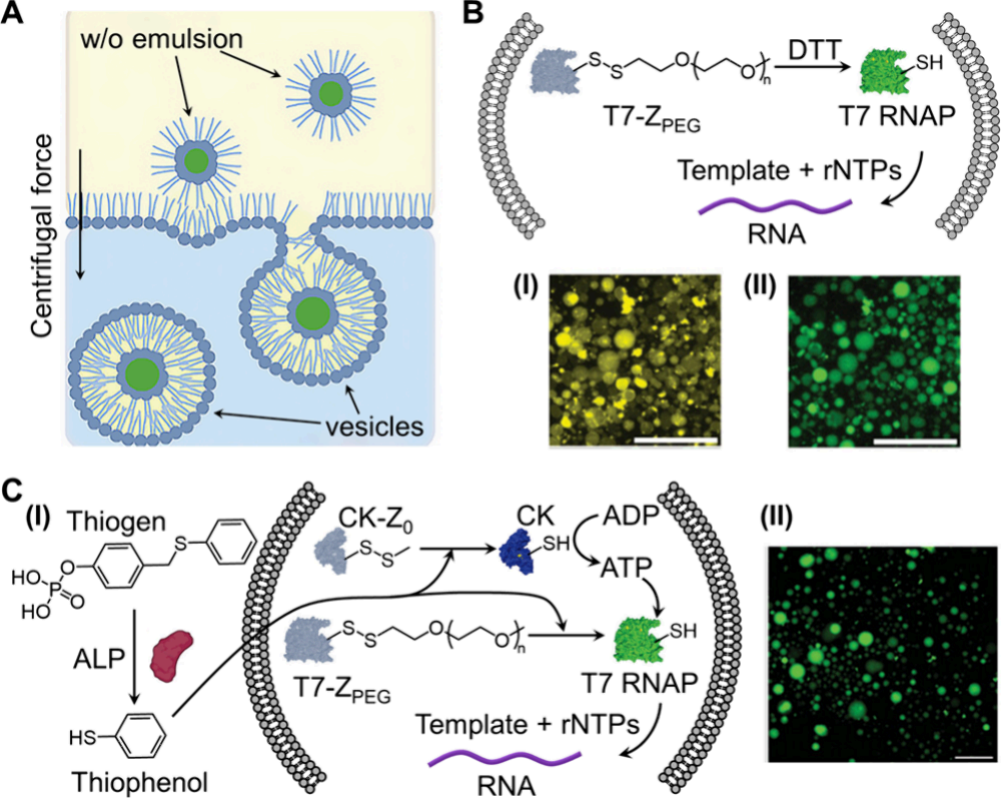
(A) Schematic assembly of loaded giant unilamellar vesicles (GUVs) using the emulsion phase transfer method. (B) Integration of the inactive redox-responsive transcription machinery composed of the disulfide-protected T7-Z_PEG_, template, and rNTPs into the GUVs, followed by the DTT redox-deprotection of the transcription machinery to synthesize the RNA product visualized by SYBR Green II staining. Panel I: Fluorescence microscopy image of the fluorophore-labeled GUV membrane. Panel II: Fluorescence microscopy image of the SYBR Green II-stained RNA product after DTT redox activation of the transcription machinery in the GUVs. (C) Panel I: Schematic illustration of the transmembrane redox activation of transcription machinery, including ALP hydrolysis of thiogen, the thiophenol-mediated deprotection of disulfide-protected CK-Z_0_, transforming ADP to ATP, and the deprotection of T7-Z_PEG_ to transcribe the RNA product. Panel II: fluorescence microscopy image of the SYBR Green II-stained RNA product upon transmembrane activation of transcription in GUVs shown in panel I. Scale bars, 100 μm. Adapted with permission from ref 51. Copyright 2023 Wiley-VCH GmbH.

The switchable reconfiguration of DNA frameworks conjugated to transcription templates was broadly used to emulate the switchable transcription factor ON/OFF modulation of native transcription processes. Diverse transcription templates modified with reconfigurable blockers, such as T·A-T triplexes, G-quadruplexes, or photoisomerizable trans-azobenzene-stabilized duplexes coupled to transcription templates, were used to reversibly switch the dynamic operation of ON/OFF transcription modules in homogeneous aqueous phases.^34^ This concept was applied to develop a triggered G-quadruplex-functionalized transcription machinery in hydrogel microcapsules as protocells.^34^ Calcium carbonate (CaCO_3_) microparticles were loaded with a transcription template composed of a promoter (4)-functionalized tetrahedra nanostructure hybridized with (5), as well as T7 RNAP. The particles were coated with the positively charged poly­(allylamine hydrochloride) (PAH), followed by the linkage of an initiator fuel strand (6). Subjecting the particles to two polymer carboxymethyl cellulose (CMC) chains, P_A_ and P_B_ functionalized with pre-engineered hairpin tethers H_A_ and H_B_, undergoing initiator (6)-fuel hybridization chain reaction (HCR), resulted in the coating of the transcription module-(4)/(5)-T7 RNAP-loaded microparticle cores with a hydrogel film cross-linked by the cross opened hairpin duplex H_A_/H_B_. The subsequent etching of the CaCO_3_ cores with EDTA resulted in 3 μm-sized hydrogel microcapsules loaded with the transcription module, Figure 5A. The relatively high molecular weight of the DNA tetrahedra-modified template, engineered to transcribe the MG aptamer, and T7 RNAP prohibited leakage from the microcapsules yet allowed the diffusion permeation of low-molecular-weight agents across the microcapsule boundaries. Accordingly, subjecting the transcription machinery-loaded microcapsules to the rNTPs and MG allowed the intracapsular operation of the transcription machinery, leading to the formation of the fluorescent MG-RNA aptamer product, Figure 5B. The transcription template was pre-engineered to reconfigure in the presence of Sr^2+^ ions into a G-quadruplex acting as a blocker unit for the transcription process. Accordingly, the permeation of Sr^2+^ across the microcapsule boundaries blocked transcription machinery. Moreover, subjecting the microcapsule to kryptofix [2.2.2] (KP) separated the Sr^2+^ ions from the G-quadruplex blocker unit, reactivating the transcription machinery, Figure 5B. The dynamic ON/OFF switchable modulation of the transcription machinery was then followed by the temporal fluorescence output of the MG-RNA aptamer, Figure 5C. The Sr^2+^/KP effects on the transcription machinery were supported by confocal microscopy visualization of the stepwise process by single microcapsule imaging, Figure 5D.

**Figure 5:**
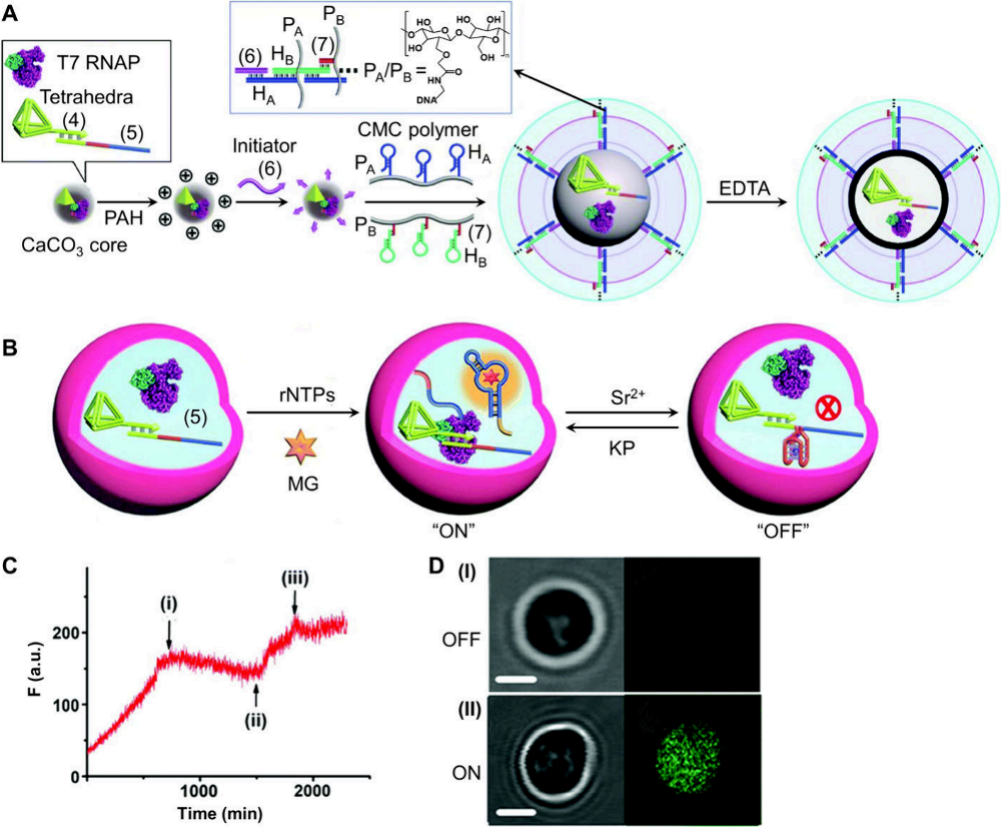
(A) Schematic synthesis of nucleic acid–based hydrogel microcapsules loaded with a transcription machinery composed of the DNA tetrahedra-modified template and T7 RNAP. (B) Schematic activation of the transcription machinery synthesizing the fluorescent MG-RNA aptamer by auxiliary added rNTPs and MG, and switchable OFF and ON operation of the transcription machinery by adding Sr^2+^/KP. (C) Switchable fluorescence intensities upon the Sr^2+^/KP-triggered transcription of the MG-RNA aptamer. At time point (i), Sr^2+^ ions were added to the system. At point (ii), KP was added to the system. At point (iii), Sr^2+^ ions were re-added to the system. (D) Confocal bright-field and fluorescence images of panel I – switched OFF transcription machinery in microcapsules treated with Sr^2+^ ions, and panel II – switched ON transcription machinery in microcapsules treated by KP. Scale bars, 1 μm. Adapted from ref 34. Available under a CC-BY 3.0 license. Copyright 2022 The Authors.

This section highlights recent advances demonstrating the integration of stimuli-responsive transcription machineries within protocell assemblies. Various external stimuli, such as light, pH, redox, and metal ions/ligands, were employed to trigger the operation of the transcription machineries. Nevertheless, all of the examples are limited to a single transcription pathway. In contrast, natural systems develop cascaded, gated, and feedback-regulated transcription networks to achieve functional complexity and adaptive behavior. Hence, future efforts to address the engineering of transcription machineries of enhanced complexity in protocells are expected. Although such advanced transcription circuits were realized in homogeneous solutions,^53,54^ their precise and spatially controlled integration into protocells remains a significant challenge. One potential strategy to address this issue involves the controlled fusion of liposomes^55^ carrying distinct transcription machineries into a single, multifunctional liposomal protocell. Moreover, in native cells, phase-separated organelles play key roles in controlling and regulating transcription processes.^56^ Recent progress in developing the phase-separated organelle-like structures within protocell assemblies^57,58^ opens the possibility of designing of synthetic organelles capable of intercommunication and dynamic regulation of embedded transcription machineries. These developments could mark a critical step toward constructing life-like protocells with programmable, adaptive gene expression systems.

## 3 Temporally Modulated Transcription Machineries in Protocells

Temporal modulation of transcription machineries in homogeneous aqueous phases has been addressed by diverse reaction modules driving the transient operation of transcription processes^54,59^ and their applications for transient DNAzyme-guided transformations,^53^ temporal coagulation of thrombin,^60^ and the assembly and disassembly of DNA nanotubes^61^ or dimmers.^62^ Besides, dynamic bistable transcription reaction modules in solution,^63,64^ oscillatory transcription modules in solution,^65^ and their applications for operating as oscillator DNA-based machines (tweezers)^66^ or the oscillatory assembly and disassembly of nanostructures^67^ were demonstrated. The integration of dynamically modulated transcription machineries into protocell assemblies is, however, scarce and involves challenging issues including the assembly of the networks/circuits in the protocell assemblies and the imaging of the dynamic transcription machineries in individual protocells versus the phenomena in collective ensemble of protocells.

In one example,^35,65,66^ Figure 6A, panel I, an oscillatory transcription machinery operating in a homogeneous aqueous phase was demonstrated. The system includes a transcription template (T_1_) that is activated by a promoter, A_1_. T7 RNAP-induced transcription yields RNA rA_1_ as the product, which displaces the auxiliary duplex A_2_/I_2_ to generate the duplex rA_1_/I_2_ and A_2_. The displaced strand, A_2_, acts as a promoter for the second transcription template, T_2_, resulting in the RNA transcription product, rI_2_, which displaces A_1_ from active transcription machinery T_1_/A_1_, deactivating transcription template T_1_ by forming the duplex A_1_/rI_2_. Since RNase H is always present in the bifunctional transcription modules, it stimulates the degradation of rA_1_ in the duplex rA_1_/I_2_. The released I_2_ then displaces A_2_ from transcription template T_2_/A_2_, leading to the deactivation of module T_2_. The concomitant RNase H-stimulated degradation of rI_2_ within the duplex A_1_/rI_2_ releases A_1_, which reactivates the transcription module T_1_/A_1_. Thus, the synchronized operation of the two coupled transcription modules results in the temporal oscillatory formation and depletion of either of the two operating transcription machineries. By labeling the integrated transcription template T_2_/A_2_ with a fluorophore/quencher pair, the oscillatory transitions of the transcription template were probed by the fluorescence intensity changes of the template T_2_, Figure 6A, panel II. Evidently, the amplitudes and frequency of the oscillations were controlled by the concentrations of T7 RNAP, rNTPs, and RNase H, and the rhythm of oscillation demonstrated a temporal dissipative decay due to the depletion of rNTPs. The oscillatory transcription module shown in Figure 6A was introduced into water-in-oil microemulsion droplets as protocell assemblies,^35^ Figure 6B. The sizes of the droplets were found to affect the oscillatory machinery within them. As the droplets were smaller, the period of oscillation was longer, Figure 6C, panel I, and the amplitude of oscillation was higher, panel II. While the effect of droplet size on the rhythm of oscillation is not fully understood, partial deactivation of RNase H by the protocell environment could be a contributing factor. The sizes of the microemulsion droplets are controlled by the surfactant/water content ratio, which affects the formation of the water-in-oil microemulsion droplets. Although the RNase H concentration in the water comprising the droplet is identical, higher concentrations of surfactant stabilizing smaller droplets might have an inhibitory effect on RNase H, leading to a lower catalytic activity that controls the rhythm of the oscillatory machinery. Thus, auxiliary effects of the protocell composition and structure could impact the catalytic activities of RNase H operating temporal transcription machinery in confined media.

**Figure 6:**
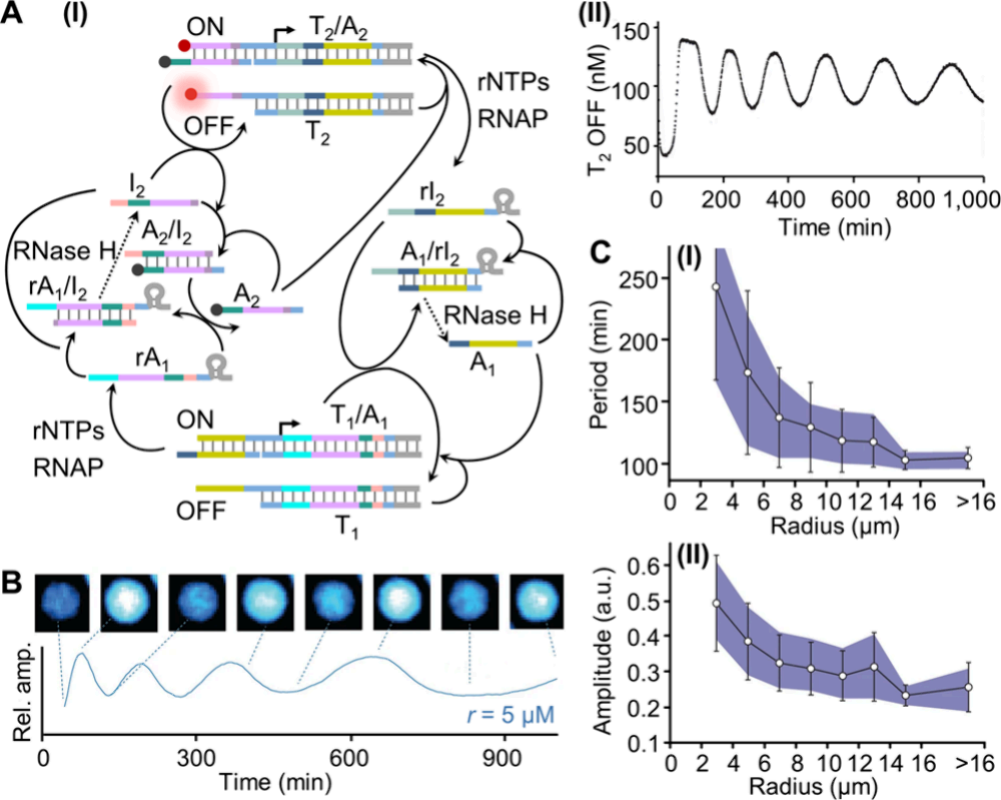
(A) Panel I: Schematic operation of an oscillatory transcription circuit consisting of two negative-feedback transcription switches. Panel II: Oscillatory temporal concentrations of the inactive fluorescent template T_2_ upon the operation of the circuit shown in panel I. (B) Top: Epifluorescence microscopy images of the transcriptional oscillator in microemulsion droplets. Bottom: Temporal fluorescence intensities in the oscillating droplet. (C) Dependence of the mean oscillation period (panel I) and corresponding amplitudes (panel II) on microdroplet radius. Adapted with permission from ref 35. Copyright 2014 Springer Nature Limited.

Emulating the dynamic assembly and disassembly of motor filaments in native cells, the transient assembly of DNA nanotubes in protocells was challenged. The transient transcription machinery-guided temporal formation and depletion of DNA nanotubes were primarily established in a homogeneous aqueous phase,^61^ Figure 7A. A reaction module consisting of an inactive tile X, composed of four complementary strands (S_1_, S_2_, S_3_, and S_4_, with strand S_1_ labeled with the Cy3 fluorophore), was mixed with a transcription template, T7 RNAP, and RNase H. Subjecting the system to rNTPs activated the transcription machinery to yield RNA (R_5_) as the product. The transcribed RNA bridged the toehold, single-stranded domains of S_2_/S_3_, and the resulting toehold tethers m’/n’ hybridized with the tethers m/n associated with strand S_4_, initiating the polymerization of the tiles into nanotube structures. The concomitant RNase H-induced degradation of the RNA strand bridging the nanotube framework led to the degradation of the nanotubes and transient recovery of the inactive reaction module. The formation and dissipative depletion of the temporally generated nanotubes were followed by fluorescence confocal imaging. This transcription machinery-guided formation of DNA nanotubes was then integrated into water-in-oil microemulsion droplets as a protocell system,^68^ Figure 7A, panel II. Imaging the transient formation and depletion of the DNA nanotubes in single droplet systems, as well as the statistic collective evaluation of the nanotubes in the droplets, is challenging due to the dynamic movement of the nanotubes in the droplets. Accordingly, sophisticated optical methods were adopted to overcome these difficulties and image the temporal formation of the DNA nanotubes in the droplets. Figure 7B depicts the temporal polymerization of two complementary DNA tiles in the droplets in the absence of transcription machinery by following the temporal pixel brightness of a single droplet. At time t = 0, a symmetric pixel brightness distribution was observed. As polymerization proceeded, an asymmetric distribution of pixel brightness was observed, resulting from the clustering of nanotubes. Analyzing the histogram of pixel brightness in the droplet using the Python Pandas Library, the skewness and kurtosis parameters of the temporal distribution of pixel brightness for single droplet were evaluated, Figure 7B, panel II. This method was then applied to track the transient transcription machinery-guided formation and depletion of DNA nanotubes in the droplets, Figure 7C. While at short time intervals a symmetric distribution of pixel brightness was observed, as the polymerization of DNA nanotubes progressed, an asymmetric distribution of pixel brightness was observed, consistent with the formation of nanotubes. The depletion of asymmetry in the pixel brightness distribution was observed at longer time intervals due to the RNase H-induced separation of nanotubes into a homogeneous distribution of tiles in the droplets. By analyzing the collective assembly of droplets, the temporal skewness and kurtosis associated with the distributions of pixel brightness provided a quantitative evaluation of the dissipative transcription machinery-driven formation and dissociation of DNA nanotubes.

**Figure 7:**
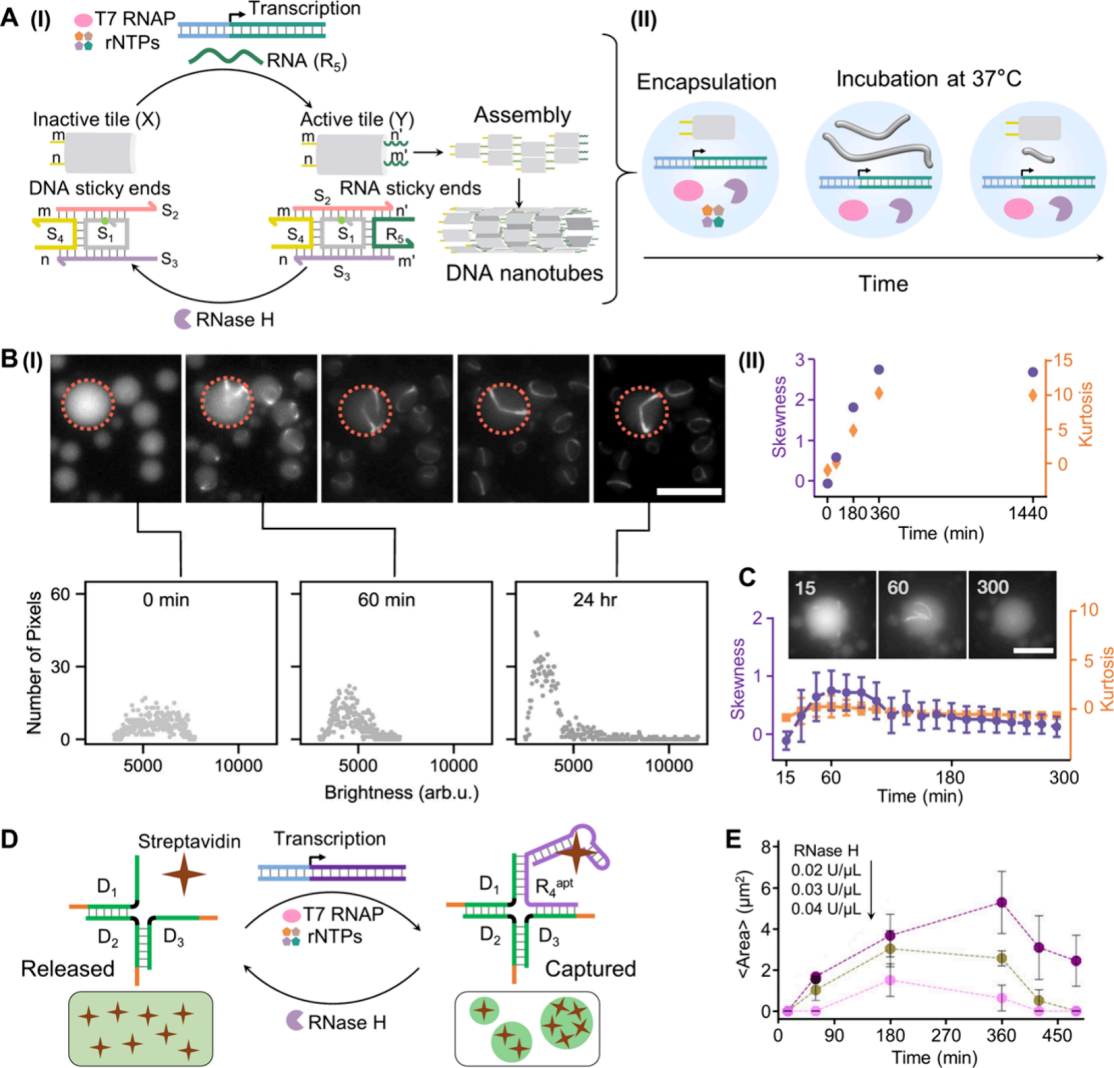
(A) Panel I: Schematic operation of a transient transcription machinery guiding the transient assembly and disassembly of DNA-RNA nanotubes. Panel II: Encapsulation of the transcription machinery in microdroplets as confined protocells for the transient transcription-guided synthesis of DNA-RNA nanotubes. (B) Panel I: Top, time-dependent fluorescence microscopy images of single microdroplets loaded with DNA tiles functionalized with two complementary sticky end strands, allowing the temporal oligomerization of the tiles into nanotube assemblies. Bottom, distribution of pixel brightness across single droplets at respective time intervals. Panel II: Skewness (purple dots) and kurtosis (orange diamonds) values corresponding to the temporal clustering of the nanotubes. (C) Temporal skewness (purple) and kurtosis (orange) values corresponding to single droplets in which the transcription machinery guided the assembly and disassembly of the DNA-RNA nanotubes. Scale bar, 20 μm. Adapted from ref 68. Available under a CC-BY license. Copyright 2021 The Authors. (D) Schematic representation of transient transcription machinery-guided transient, dissipative assembly of and disassembly of DNA-RNA condensates, accompanied by the capture and release of streptavidin into host condensates. (E) Time-dependent changes in the average condensate area at different concentrations of RNase H. Adapted from ref 69. Copyright 2024 American Chemical Society.

In addition, the transcription machinery-guided dynamic formation and depletion of DNA-RNA condensates, accompanied by the temporal uptake and release of a protein load, were demonstrated,^69^ Figure 7D. A star-like framework, consisting of three partially complementary strands (D_1_/D_2_/D_3_) with extended single-strand toehold tethers, acted as the core unit for assembling the condensates. In the presence of a DNA template, T7 RNAP, NTPs, and streptavidin (SA), the transcribed RNA was predesigned to include the SA aptamer sequence extended by RNA domains complementary to single-stranded D_3_ and D_4_ of the DNA star framework. The transcription process produced RNA that bound SA through the aptamer units, resulting in the formation of SA-captured, phase-separated condensates composed of cross-linked DNA-RNA framework units. In the presence of coadded RNase H, the hybridized RNA domains were cleaved, leading to the disassembly of the condensates and the release of the captured SA. The temporal dynamic formation and depletion of the condensates, along with the accompanying uptake and release of the SA load, were monitored by labeling the DNA framework and the SA load with two different fluorophores. The dynamic formation and depletion of the condensates were controlled by the concentration of RNase H, Figure 7E.

This section highlights recent progress in integrating transient or oscillatory transcription machineries and circuits into protocell assemblies. In biological systems, such transient or oscillatory transcription processes are typically governed by transcription factors that temporally interact with transcription machineries to orchestrate transient or rhythmic gene expression. However, the realization of analogous, transcription factor-mediated, temporally controlled transcription machineries even in homogeneous solutions is unprecedented. Moreover, current protocell models demonstrating transient or oscillatory transcription are primarily limited to transcriptional activity alone, lacking the subsequent coupling to significant translational processes that are critical for functional output. Hence, a major future challenge in protocell design lies in integrating transcription factor-guided, spatiotemporally regulated transient or oscillatory operation of transcription machineries with the compartmentalized and sequestered execution of the corresponding transient or oscillatory translation processes.

## 4 Dynamic Transcription-Mediated Diffusive Communication of Protocells

The operation of multicellular interacting systems in nature represents a major step in evolution of life.^70,71^ Diverse biochemical transformations in living cells are interconnected and integrated through intracellular and intercellular communication,^72^ leading to collective behaviors and functionalities such as adaptation, motility, or energy consumption.^73,74^ Native intracellular and intercellular communication originates from complex spatiotemporal organization of networks and circuitries, which involves signaling, vectorial^75^ and synchronized^76^ communication, programmed exchange of information,^77,78^ and the operation of biochemical machineries in microcompartmentalized cellular environments, leading to collective cellular phenomena.^79–83^ Recent advances in DNA nanotechnology and the construction of protocell model systems provide unique tools to construct biomimetic model systems that emulate native intracellular and intercellular communication processes. In the present section, the integration of transcription machineries into microcompartmentalized protocell assemblies and the dynamic transcription-guided protocellular communication pathways will be addressed.

In one example,^84^ Figure 8, a protocell, consisting of a DNA microcompartmentalized condensate that included a spatially localized transcription machinery, was assembled. The operation of the transcription machinery led to the synthesis of an RNA product that included structural information for guided spatial translocation across the compartmentalized domains. The DNA condensate protocell particles were assembled from four-arm DNA junctions cross-linked by hydrophobic, cholesterol-stabilized nucleic acid micelles. One of the DNA arms, comprising the cross-linked DNA core units, included an overhang tether l to which an auxiliary sequence-specific barcode strand b was hybridized, Figure 8A. The DNA condensate provided a functional protocell framework for the competitive compartmentalization by “invading strands”, which were composed of variable lengths of hybridization strands (r and c) complementary to the barcode domain of b. Compartmentalization of the condensate frameworks was guided by the diffusive features of the invading strands and the hybridization efficiencies of the respective strands with the barcode domain. Rapid invasion of the condensate core by the short, rapidly diffusing invader strand r was accompanied by slower invasion of the condensate by the longer, slower-diffusing invader strand c (which displaces the shorter strand r due to enhanced hybridization stability). This is exemplified in Figure 8B. To assemble a functional transcription machinery within the protocell compartments, a transcription template, composed of a duplex r/t with r labeled with Alexa 647N, was hybridized to the barcode domain of b in the condensates. Subsequently, an auxiliary invading strand c was employed to displace the exterior transcription templates associated with the condensates, yielding bicompartmentalized condensates, where the core contained the transcription template, and the shell compartment included barcodes hybridized with tether strands p. The dynamic competitive invasion of the condensates was blocked by the hybridization of the “stopper” strand s with the auxiliary solubilized invading strands r and c, resulting in equilibrated and compartmentalized condensates. As shown in Figure 8C, subjecting the porous condensates to T7 RNAP, rNTPs, and the 3,5-Difluoro-4-hydroxybenzylidene imidazolidinone (DFHBI) ligand activated the transcription machinery in the core domains of the condensates. The transcription template t was engineered to transcribe the broccoli RNA aptamer, and the tether p associated with the shell compartments of the condensates was designed to hybridize with a domain of the broccoli aptamer. The freely diffusive transcribed aptamer was then translocated to the shell microcompartments. The dynamic translocation of the transcribed RNA was monitored by the formation of the fluorescent DFHBI/aptamer complex in the outer shell microcompartment, Figure 8D. This system demonstrates the intracellular communication of microcompartmentalized domains where the core domain provides the RNA signal for the receiver compartment.

**Figure 8:**
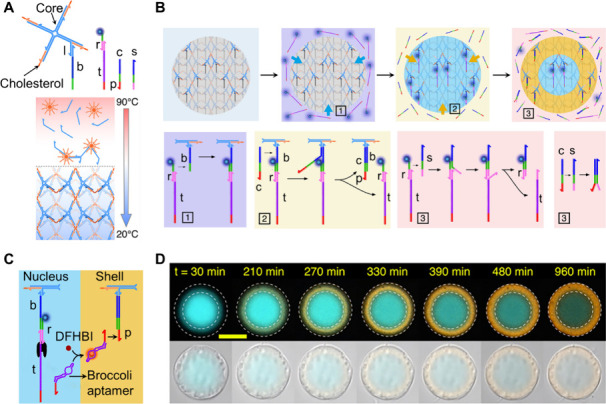
(A) Schematic structure of a four-arm DNA junction core unit for the self-assembly of a DNA condensate protocell. (B) Schematic assembly of a bicompartmentalized DNA condensate protocell by the competitive invasion of the condensate with different lengths of invading strands, with the core domain loaded with the transcription template composed of the duplex r/t, and the shell compartment functionalized with a receiver strand p. (C) Schematic activation of the transcription machinery in the condensate, accompanied by signal transfer across the bicompartmentalized condensate. Subjecting the condensate to T7 RNAP/NTPs yields, in the core domain, the broccoli RNA aptamer that is transferred to the shell compartment through hybridization to the p strand, resulting in the accumulation of the fluorescence DFHBI/aptamer complex in the shell. (D) Temporal fluorescence changes of the bicompartmentalized condensate upon the transcription-guided formation of the RNA aptamer in the core domain and its dynamic transfer to the shell compartment. Scale bar, 15 μm. Adapted from ref 84. Available under a CC-BY 4.0 license. Copyright 2022 The Authors.

The intercommunication between protocell assemblies has been demonstrated using the interconnected, membrane-separated microdroplets,^85^ Figure 9A. Two types of interconnected microdroplets were assembled. One type of microdroplets (“sender”) included the quorum sensing signal C6-HSL (*N*-(3-oxohexanoyl)-l-homoserine lactone) and the transcription inducer arabinose. The second type of microdroplets (“receiver”) was loaded with a pLux promoter-functionalized GFP plasmid, activated by C6-HSL, and a pBAD promoter-functionalized red fluorescent protein (RFP) plasmid, activated by arabinose. In Configuration I, the “sender” and “receiver” microdroplets were connected by an intervening buffer solution-containing droplet. This configuration resulted in the selective expression of GFP, Figure 9B, panel I, indicating that C6-HSL diffused from the “sender” microdroplet, through the intervening buffer-containing droplet, to the “receiver” microdroplet, where it activated GFP expression. In Configuration II, the lipid membranes of microdroplets were functionalized with α-hemolysin (α-HL) pore-generating protein channels. This configuration allowed for the simultaneous expression of both RFP and GFP, Figure 9B, panel II. These results confirmed that the promoter signaling C6-HSL underwent the parallel diffusive crossing through the lipid boundaries while arabinose underwent permeation through the α-HL channel from the sender protocell into the receiver protocell, resulting in the protocellular intercommunication-mediated transcription and translation of the respective fluorescent proteins. The interconnection between “sender” and “receiver” microdroplets was further exemplified by the sequential signal-triggered activation of transcription machinery across an array of “receiver” droplets, Figure 9C. The sender droplet was loaded with the DFHBI ligand and labeled with a red fluorescent Atto655-nucleic acid marker. The array of receiver microdroplets were loaded with T7 RNAP/rNTPs, an inactive partial promoter/transcription template (N_1_/T_1_), and the anti-DFHBI Spinach RNA aptamer A_1_ which was blocked by the DNA promoter subunit P_1_. The diffusive permeation of the DFHBI ligand across the boundary between the interconnected “sender” and “receiver” microdroplets displaced the A_1_ aptamer, resulting in the formation of the fluorescent DFHBI/A_1_ complex and the release of promoter P_1_. The released P_1_ then binds to the incomplete N_1_/T_1_ transcription template, activating the transcription machinery to produce RNA A_1_’. The transcribed RNA A_1_’ was engineered, however, to be complementary to the A_1_ aptamer, resulting in the dissociation of the DFHBI/A_1_ complex and the release of DFHBI in the primary receiver microdroplet. The subsequent diffusive permeation of the DFHBI ligand from the primary receiver microdroplet to a second receiver microdroplet activated the transcription machinery in the second “interconnected” microdroplet, thereby establishing a chain mechanism for the sequential temporal transmission of the DFHBI signaling ligand across microdroplet boundaries. The temporal intercommunication and operation of transcription machineries across the array of microdroplets were followed by the temporal fluorescence changes of the DFHBI aptamer product, which was generated and depleted in the respective microdroplets, Figure 9D, panels I and II.

**Figure 9:**
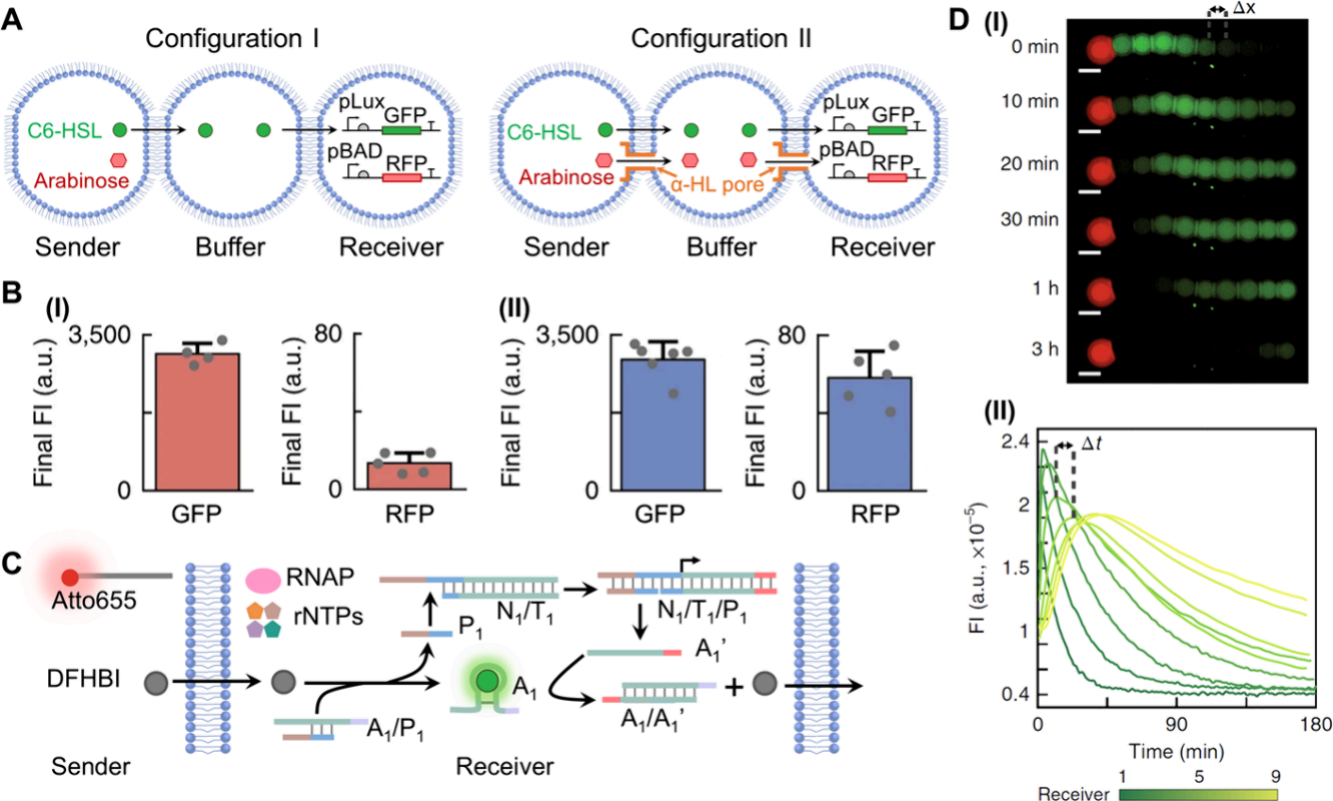
(A) Schematic intercommunication of microdroplets by transcription inducers-mediated activation of transcription and translation machineries. The “sender” microdroplets were loaded with C6-HSL and arabinose as transcription inducers, while the “receiver” microdroplets were loaded with a pLux-GFP plasmid template (activated by C6-HSL) and the pBAD-RFP plasmid template (activated by arabinose). In configuration I, where the buffer droplet lacks α-HL protein, the diffusive permeation of C6-HSL across the lipid boundaries of the three microdroplets allows only the activation of the pLux-GFP transcription/translation machinery, synthesizing the GFP. In configuration II, where the pore-generating α-HL protein is present in the buffer microdroplet, the diffusive permeation of C6-HSL and α-HL pore-guided transport of arabinose across the boundaries of the three droplets proceed, leading to the parallel expression of GFP and RFP. (B) Fluorescence intensity (FI) levels corresponding to panel I – selective expression of GFP by configuration I, and panel II – parallel expression of GFP/RFP by configuration II. (C) Signal transmission from a “sender” microdroplet protocell to a “receiver” protocell loaded with the transcription machinery reaction module, activating the chained signal transmission output to “receiver” protocells. Diffusion of DFHBI from the “sender” to the “receiver” protocell displaces the A_1_/P_1_ duplex in the “receiver” protocell, resulting in the green fluorescent DFHBI/aptamer complex and the triggered activation of the transcription machinery. The transcribed RNA displaces the DFHBI/aptamer, releasing the DFHBI signaling output and activating the chained operation of the “receiver” protocells. (D) Panel I: Temporal microscopy images following the temporal fluorescence of the DFHBI/aptamer complexes generated in the intercommunicated chain of “receiver” microdroplets. Scale bars, 200 μm. Panel II: Temporal fluorescence changes of the intercommunicated, chained, individual “receiver” microdroplets. Δt indicates the time between two pulse maxima. Adapted with permission from ref 85. Copyright 2018 Springer Nature Limited.

Moreover, the intercommunication between protocell assemblies was accomplished through a light-activated transcription/translation machinery.^86^ Figure 10A depicts the assembly of the light-activated transcription/translation machinery synthesizing the yellow fluorescent protein mVenus in a water-in-oil microemulsion droplet protocell system. The promoter within the transcription template was functionalized with the photoresponsive biotin-2-nitrobenzyl-amino-thymine bases. Binding of streptavidin to biotin led to an inactive transcription template, inhibiting its interaction with T7 RNAP. The inactive transcription template encoding for mVenus was integrated with IVTT components in a lipid-stabilized microemulsion droplet (lipid: 10% 1,2-dipalmitoyl-*sn*-glycero-3-phosphoethanolamine-*N*-[methoxy­(polyethylene glycol)-2000] in diphytanoyl­phosphatidylcholine). The lipid coating allowed interaction between droplets and enabled the 3D-printing of layered microdroplet structures, forming tissue-like assemblies. Photochemical uncaging (λ = 365 nm) of the 2-nitrobenzyl protecting groups released the streptavidin–biotin blocker units, resulting in the activation of the transcription/translation machinery and the intraprotocell or intratissue expression of mVenus, Figure 10B, panels I and II. Additionally, analogous droplets were loaded with the photoresponsive inactive transcription template encoding for α-HL and IVTT components, with the capacity of synthesizing α-HL protein upon light activation. The resulting microdroplet (“receiver”) was interconnected to the microdroplet (“sender”) loaded with the small-molecule fluorophore carboxytetramethylrhodamine (TAMRA) through boundary lipid interactions, Figure 10C. Photochemical deprotection of the transcription/translation machinery led to the accumulation of the α-HL protein at the interprotocell boundary and the formation of a pore channel, mediating the intercommunication between protocells and the transport of fluorescent TAMRA from the “sender” droplet to the “receiver” droplet. Furthermore, by inserting two microelectrodes into the protocells and applying a potential between them, directed ionic currents were demonstrated upon light-activated expression of α-HL, which were lacking in the absence of the α-HL pore, Figure 10D.

**Figure 10:**
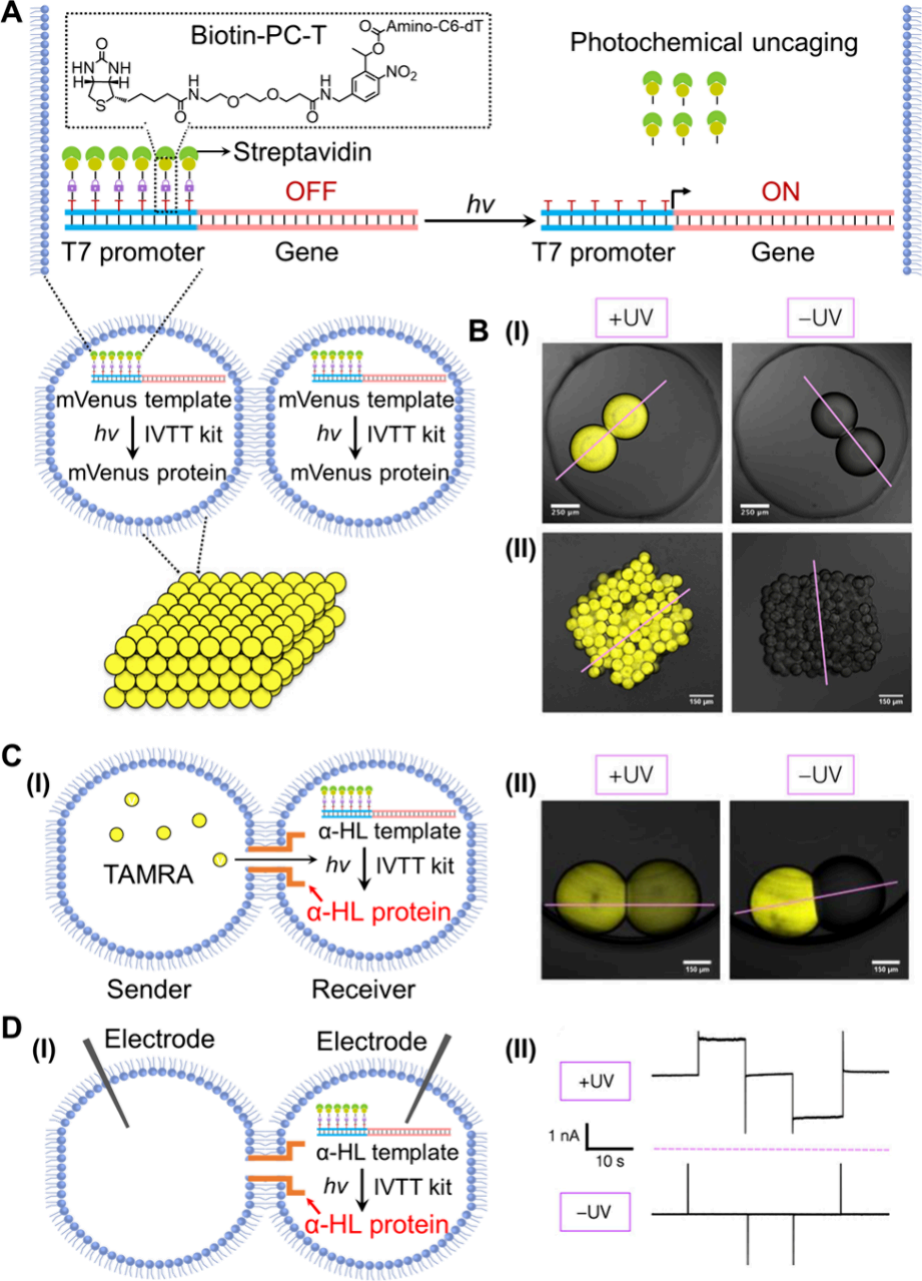
(A) Schematic integration of a photoresponsive, caged, mute promoter/gene plasmid template and the IVTT transcription/translation kit into lipid-coated water-in-oil microemulsion droplets. Photochemical deprotection of the template activates the expression of the yellow fluorescent protein mVenus in the droplets or a printed tissue-like 3D assembly of protocells. (B) Confocal microscopy images of the yellow fluorescent protein mVenus expressed in an interconnected microdroplet dimer (panel I) and the printed tissue-like 3D assembly of protocells (panel II) upon light-triggered deprotection of the transcription/translation machinery, and the control systems in the absence of light activation. (C) Panel I: Schematic of the caged α-HL transcription template/IVTT reaction module expressing the α-HL pore-generating protein upon photochemical uncaging. Integration of the expressed α-HL protein into the lipid boundary interconnecting the sender-receiver protocells facilitates the pore-assisted transport of the fluorescent TAMRA from the “sender” into the “receiver” protocell. Panel II: Confocal microscopy images of the photoactivated expression module of α-HL facilitating the α-HL pore-assisted transport of TAMRA from the “sender” to “receiver” protocells across the lipid boundary (left). Control experiments in the absence of photochemical uncaging of the expression module. (D) Panel I: Schematic design of a biprotocell electronic system, where the photoresponsive, caged, inactive α-HL transcription template/IVTT expression kit is integrated in one of the protocell dimers, and two microelectrodes are inserted in the respective dimer protocells. Photodeprotection of the expression module results in the α-HL protein integrated into the lipid boundary of the protocell dimer. Applying a potential across the two microelectrodes yields an ion current flow across the interconnected protocells. Panel II: Top – ionic currents generated upon photochemical deprotection of the expression module synthesizing α-HL. Bottom – control system employing an uncaged reaction module, upon applying potential steps across the microelectrodes. Adapted with permission from ref 86. Copyright 2016 The American Association for the Advancement of Science.

In a further example, the transcription-mediated intercommunication of two populations of proteinsomes was demonstrated,^87^ Figure 11A. The two populations of proteinsomes were loaded with streptavidin. The proteinsomes were prepared by using a water-in-oil microemulsion system consisting of aqueous microdroplets stabilized by fluorescein (FITC)-labeled bovine serum albumin (BSA)/poly­(*N*-isopropylacrylamide) (BSA-NH_2_/PNIPAAm) nanoconjugates, which acted as surfactants. Polymerization of the surfactant yielded streptavidin-loaded proteinsome-based microcapsules stabilized by protein/polymer membrane boundaries. Using a microfluidic trapping device, distinct populations of proteinsomes were assembled. While population 1 was loaded with a biotinylated and Texas Red (yellow emission)-modified inactive transcription template N_1_/T_1_, population 2 was loaded with a biotinylated Cy5 (red fluorescence)/Lowa Black quencher-functionalized transducing reporter duplex F_1_/Q_1_. The mixture of “dormant” proteinsomes revealed the FITC green fluorescent label associated with the two populations, Figure 11B, panel I, and the Texas Red yellow fluorescence associated with population 1 (panel II). Binding of the Lowa Black Quencher-labeled input I_1_ to the transcription template resulted in the activation of the transcription machinery in population 1, synthesizing the RNA product R_1_, which was accompanied by the quenching of the yellow fluorescent Texas Red. The R_1_, released from population 1, underwent diffusive permeation into population 2 and was pre-engineered to displace the reporter duplex F_1_/Q_1_ in population 2. Thus, the diffusive, transcribed RNA (R_1_)-mediated intercommunication of the two populations of proteinsomes was transduced by the temporal quenching of the Texas Red yellow fluorescence in population 1 and the concomitant switching on of the Cy5 red fluorescence associated with population 2 (panel III). The transcription-mediated intercommunication of two proteinsomes was further extended by demonstrating the transcription machinery-mediated functional activation of an auxiliary protein (deactivated Cas9, dCas9) and its guided diffusive permeation into a target proteinsome protocell, Figure 11C. The system consisted of two different populations of proteinsomes: population 3 was loaded with a biotinylated inactive transcription template N_2_/T_2_, and population 4 was loaded with a duplex unit composed of a fluorophore (Cy5)-modified strand F_2_, hybridized with two strands Q_2_ and Q_3_, where Q_2_ is modified with a quencher label and Q_3_ is functionalized with a biotin unit (the duplex F_2_/Q_2_+Q_3_ acts as a transducer reporter for the intercommunication process). The deactivated auxiliary protein dCas9 was present in the bulk reaction medium. In the absence of input I_2_, population 3 lacked any fluorescence, and similarly, population 4 lacked fluorescence due to the quenching of Cy5 by Q_2_. Subjecting the mixture of proteinsomes to the Cy3-modified input strand I_2_ resulted in diffusive permeation of I_2_ into population 3. As I_2_ was pre-engineered to act cooperatively as a promoter unit of the transcription template, the transcription machinery in population 3 was activated, yielding the RNA product R_2_. The released R_2_ from population 3 was engineered, however, to bind to dCas9, leading to the formation of an activated dCas9/R_2_ supramolecular complex. Diffusive permeation of the active dCas9/R_2_ complex into population 4 resulted in the release of Q_2_ from the reporter unit and the switched ON of Cy5 fluorescence in supramolecular complex dCas9/R_2_/F_2_/Q_3_. The transcription machinery-guided intercommunication of the two proteinsomes, resulting in the targeted delivery of activated dCas9/R_2_ into population 4, was then measured by the temporal fluorescence changes of Cy3 in population 3 and Cy5 in population 4, Figure 11D.

**Figure 11:**
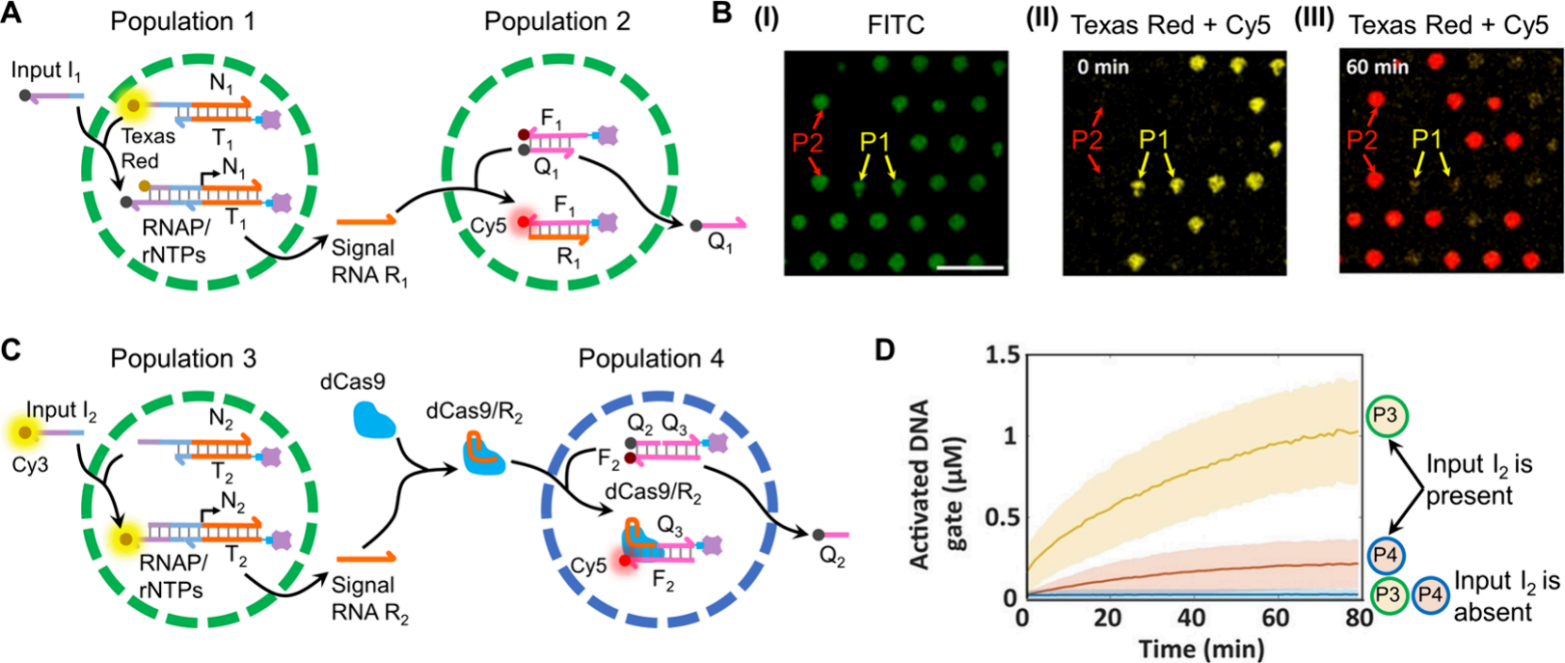
Dynamic transcription-mediated diffusive intercommunication of proteinsome-based protocells. (A) Schematic of diffusive RNA intercommunicated proteinsome populations P1 and P2. FITC (green fluorescence)-labeled proteinsome P1 is loaded with the Texas Red-labeled inactive transcription template N_1_/T_1_, exhibiting yellow fluorescence. FITC (green fluorescence)-labeled proteinsome P2 is loaded with the Cy5/Lowa Black quencher-modified duplex F_1_/Q_1_. The input I_1_-driven operation of the transcription machineries in P1 yields the transcribed RNA R_1_ that diffusively permeates into P2, where the displacement of F_1_/Q_1_ switches ON the fluorescence of F_1_. (B) Confocal fluorescence micrographs of: panel I – proteinsomes P1+P2 through FITC channel, panel II – P1+P2 through Texas Red and Cy5 channels, prior to application of input (only yellow emission of Texas Red visible), panel III – P1+P2 after application of input for 60 min through Texas Red and Cy5 channels (only red emission of Cy5 visible). Scale bar 100 μm. (C) Schematic of dCas9/R_2_-mediated intercommunication of proteinsomes P3 and P4. P3 includes the inactive transcription template N_2_/T_2_ and P4 includes the duplex probe F_2_ (Cy5)/Q_2_ (quencher)+Q_3_. dCas9 is present as an auxiliary constituent in the bulk solution. Triggering the mixture of P3 with the Cy3-labeled input I_2_ activates the transcription machinery, transcribing RNA R_2_. The escaped R_2_ forms the dCas9/R_2_ complex that diffusively permeates into P4, resulting in the displacement of Q_2_ and the fluorescent dCas9/R_2_/F_2_/Q_3_ supramolecular structure. (D) Time-dependent concentrations of the input-activated transcription template in P3 (yellow line) and the activated dCas9 probe in P4 (red line) upon the I_2_-triggered intercommunication of P3 and P4. Adapted from ref 87. Available under a CC-BY license. Copyright 2022 The Authors.

This section introduced the application of various transcription machinery-loaded protocell assemblies, including DNA condensates/coacervates, microdroplets, and proteinsomes, as functional reaction reservoirs for facilitating intracellular or interprotocellular chemical transformations. These transcription machineries were employed to generate RNA strands capable of mediating communication either between compartmentalized domains in a single protocell or across neighboring protocells. However, in all reported systems, only a single transcription machinery was operative. Looking forward, the design of protocells incorporating the intercommunication between layered and cascaded transcription machineries, featuring gated, temporally controlled, or feedback-regulated operation, represents a desirable and challenging direction. Moreover, establishing communication between synthetic protocells and native cells offers a promising avenue, particularly in the context of biomedical applications. The transfer of mRNA strands synthesized within protocells into native living cells could enable the intracellular operation of synthetic therapeutic agents, e.g., ribozymes or regulatory RNAs, thereby opening new frontiers in synthetic biology and targeted therapy.

## 5 Conclusions and Outlook

Diverse protocell compartments for operating dynamic transcription machineries were introduced, including liposomes, an NMOFs-loaded membrane, microcapsules, DNA condensates/coacervates, microdroplets, and proteinsomes. Different transcription-guided reaction circuits within these protocell assemblies were discussed, including transcription machineries operating in confined protocell compartments, switchable and dynamically modulated transcription circuits, and transcription-guided, signal-driven intercommunication between protocell assemblies. While substantial progress and advances in integrating transcription machineries into protocells were demonstrated, significant future challenges are ahead of us.

The integration of transcription modules revealing enhanced complexities within protocell compartments is certainly a challenging path. These could include bistable transcription circuits and gated, cascaded, and temporally modulated intraprotocell transformations. Moreover, designing other transcription module-loaded protocells, such as polymersomes or dendrosomes, and particularly achieving subcompartmentalization of protocells with transcription-guided, signal-triggered, vectorial spatiotemporal transformations across the subcompartments, are important issues to address. Additionally, evaluation of the effects of size, shape, and material composition on the functions of protocell compartments is crucial for advancing this field.

Mimicking native cell functions, the signal intercommunication of protocells, is essential. This will require the sequential and parallel signal-guided assembly of protocells operating via cascaded, gated, and branching mechanisms. As these chain reactions involve signal-transfer, weakening of the signals and leakage phenomena are anticipated to be faced; thus, the development of cooperating feedback amplification mechanisms is important. At present, diffusive signal communication is employed to propagate and control the processes, yet future efforts applying supramolecular interprotocell interactions will play key roles in the effective information transfer operating the ensemble of protocells. Moreover, the transcription-guided operation of protocell assemblies will require precise spatiotemporal control of the systems. Switchable, transient, and oscillatory transcription circuits within protocell assemblies are anticipated to gain growing interest. Auxiliary physical signals, such as light, electrical, magnetic, ultrasound, and chemical stimuli, including pH, ions, and specific ligands, will play important roles in driving these processes. Particularly, nanotechnology provides unique tools to address and activate subcompartments of protocell compartments, facilitating the operation of cascaded networks. Furthermore, extending the dynamic transcription processes within protocells to include protein translation processes presents another key challenge for future research.

Finally, potential applications of protocells should be considered. Probing the protocell responses to auxiliary triggers such as ions, biomarkers, or ligands provides a means to apply the protocells as functional sensing^88^ and multiplexed imaging compartments. Transcription machinery-loaded protocells are expected, however, to serve as effective carriers and delivery vehicles for therapeutic applications. Recent advances reported on the light-triggered fusion of drug-loaded protocells with native cells and the accompanying drug release,^89^ suggesting protocells as effective drug delivery vehicles. Moreover, in vitro studies have demonstrated dynamic transcription-guided modulation of DNAzyme functions,^53^ thrombin-catalyzed blood clotting,^60^ and light-stimulated operation of the CRISPR/Cas9 machinery for gene editing.^90,91^ Integrating these and other circuitries into protocells, followed by fusion with native cells, is anticipated to provide a versatile means for the spatiotemporal delivery of diverse therapeutic agents, particularly gene editing and repair machineries. In this context, the incorporation of transcription machineries within protocell assemblies offers an effective means to protect newly synthesized RNA from enzymatic degradation prior to its delivery into native cells.
